# Extracellular vesicles: Targeting the heart

**DOI:** 10.3389/fcvm.2022.1041481

**Published:** 2023-01-10

**Authors:** Xin Yin, Li-Hong Jiang

**Affiliations:** ^1^Faculty of Life Sciences and Technology, Kunming University of Science and Technology, Kunming, China; ^2^Department of Ultrasound, The Affiliated Hospital of Kunming University of Science and Technology, Kunming, Yunnan, China; ^3^The First People’s Hospital of Yunnan, Kunming, Yunnan, China

**Keywords:** extracellular vesicles, cardiovascular diseases, target delivery, myocardial infarction, exosome (vesicle)

## Abstract

Cardiovascular diseases rank the highest incidence and mortality worldwide. As the most common type of cardiovascular disease, myocardial infarction causes high morbidity and mortality. Recent studies have revealed that extracellular vesicles, including exosomes, show great potential as a promising cell-free therapy for the treatment of myocardial infarction. However, low heart-targeting efficiency and short plasma half-life have hampered the clinical translation of extracellular vesicle therapy. Currently, four major types of strategies aiming at enhancing target efficiency have been developed, including modifying EV surface, suppressing non-target absorption, increasing the uptake efficiency of target cells, and utilizing a hydrogel patch. This presented review summarizes the current research aimed at EV heart targeting and discusses the challenges and opportunities in EV therapy, which will be beneficial for the development of effective heart-targeting strategies.

## Introduction

Cardiovascular diseases (CVDs) rank the highest incidence and mortality worldwide (1–4). It is estimated that the number of deaths caused by CVD is 17.3 million every year, and this figure is expected to surpass 23.6 million by 2030 (5). Coronary artery disease, especially myocardial infarction (MI), is the most common type of CVDs and has become a major contributor to high morbidity and mortality (6). Myocardial infarction causes a large amount of myocardium loss, extracellular matrix disorder, and scar formation (7, 8). Continuous loss of numerous cardiomyocytes finally leads to ventricular remodeling and heart failure (9). Although the clinical adoption of drug therapy, thrombolysis, coronary stent placement, and coronary artery bypass grafting has improved the survival rate for patients with MI, these methods fail to effectively reduce the myocardial injury caused by ischemia/reperfusion and restore heart function (10).

Cell-based therapies, such as mesenchymal stem cells (MSCs), have shown great potential for the treatment of MI. However, the clinical application of cell therapies in MI is somehow disappointing: It is reported that compared to traditional treatment, stem cell therapy did not reduce the risk of rehospitalization for heart failure or the combined risk of death, neither resulting in any improvement in heart function (11). In addition, there are some unexpected side effects brought by cell therapies, for example, MSCs may transform into fibroblasts and thus aggravate myocardial fibrosis (12). Moreover, MSCs might trigger severe calcification in the heart (13). Recent studies suggest that cell-mediated paracrine signaling, rather than differentiation, is the main mechanism of cell-based therapy, and extracellular vesicles (EVs) played an important role in this process (14, 15).

Extracellular vesicles are nano- to micron-sized vesicles ranging from 30 to 1,000 nm in diameter, which were secreted by almost all types of cells. The International Society for Extracellular Vesicles (ISEV) has recommended that particles with lipid bilayers that have been released from cells are referred to as EVs (16). Generally, EVs are classified into three subgroups: exosomes, microvesicles, and apoptotic bodies. Due to the overlap in physicochemical properties and lack of definitive molecular markers, it remains challenging to accurately distinguish EV subtypes (17–20). Hence, it is preferable to use the generic term “extracellular vesicles” rather than exosomes. The therapeutic function of EVs can be attributed to their capacity to deliver curative substances to the recipient cells. These substances include non-coding RNAs, mRNAs, proteins, and mitochondria (21–24). The contents of EVs have the potential to trigger a variety of cardioprotective effects, including increasing the survival of cardiomyocytes and endothelial cells, suppressing oxidative stress and inflammatory response, reducing scar formation and left ventricular remodeling, and stimulating post-ischemic angiogenesis (22–24). Compared with conventional cytotherapy, EVs show several advantages as follows: (1) EVs transplantation is cell-free therapy, which is safer than cell transplantation due to that EVs present a low risk of tumorigenicity or trigger an arrhythmogenic response, as well as low immunogenicity and high biocompatibility (25–29); (2) the condition required for EVs storage and transportation is less harsh than cells, and the components of EVs remain stable at −20 to −80°C for a long time without degradation (30–33); (3) in the treatment of MI, MSCs transplanted at the site of myocardial infarction region cannot withstand the ischemic and hypoxic microenvironment and end up mass dying. On the contrary, EVs have no such defect (34); (4) EVs can be loaded with therapeutic cargo and targeted delivery to the lesion zone (26). In terms of drug delivery, viral vectors can transfer gene cargo efficiently and overcome cellular barriers. However, the broad use of viral vectors is unfortunately constrained due to their intrinsic drawbacks, which include tropism, safety issues, production challenges, and a restricted capacity for packaging. Non-viral vectors, such as lipid nanoparticles and liposomes, have been shown to be flexible, are easy to modify, and have high gene packaging capacity. In contrast to natural vectors, artificial vectors are much simpler, so they have difficulties precisely targeting and crossing multiple barriers *in vivo* (35). EVs can be consider as “nature’s lipid nanoparticles,” as they originate from biological systems (36). Compared with cells and other drug-delivery vehicles, EVs have several advantages because of their natural structure. For example, artificial nanoparticles are easily removed from the bloodstream, whereas natural EVs could avoid this clearance mechanism to a great extent. Furthermore, EVs’ cell-derived membranes granted them minimal toxicity and immunological covertness (37). Lastly, EVs inherit functions of their source cells, as MSC-derived EVs and MSCs are reported to yield similar therapeutic benefits (34, 38). A large number of preclinical experiments have proven that EVs are a promising “medicine” in tackling cardiovascular disease (34, 38–59). Meanwhile, the side effects of EV-based treatments have scarcely been reported. MSCs contribute to tissue repair and regeneration mainly through EV-mediated paracrine, which makes them an ideal source for EV production. Both *in vitro* and *in vivo* studies have demonstrated that MSCs are high-yielding sources of EVs, and MSC-derived EVs can improve cardiac functions in ischemia–reperfusion injury (26, 60).

To effectively translate EV-based therapies from bench to clinic, how to improve the short plasma half-life and low targeting efficiency of EVs is pivotal. In most animal experiments, EVs were administered *via* intramyocardial or intracoronary injection (34, 58, 59). These are undoubtedly efficient methods for EV delivery but are unsuitable for clinical practice, as the processes are invasive, complex, and high-risk practice. In contrast, intravenous delivery is reproducible and technically easier, which make it more preferred due to that patients with MI can poorly tolerate repeat invasive operation. However, this approach is hampered by off-target binding and poor accumulation of EVs in the cardiac tissue. Since it has been revealed that after intravenous administration, EVs are distributed largely to the liver, the lungs, the kidneys, and the spleen (61–65).

It is reported that less than 10% of EVs administrated by intravenous injection are finally absorbed by an uninjured heart (22); therefore, to counteract non-specific delivery, an extra dose of EVs is needed. EVs delivered by a systemic route are barely absorbed by the interstitial myocardium because the myocardial capillary endothelium is about 4 nm in diameter and is continuous with tight junctions, which facilitates the transfer of blood-borne substances but limits the movement of EVs (66, 67).

Owing to EV off-target effects and the low production yield, there is an urgent need to improve the targeted delivery of EVs to cardiomyocytes to accelerate the clinical application of EV therapy. The distribution of EVs can be influenced by many factors, including delivery mode, EVs’ size, injected dose, membrane components of EVs, as well as the type of EV receptor cells and donor cells (68–73). However, the exact mechanisms underlying EV biodistribution and uptake *in vivo* are still unclear so far. In this review, we summarize the study’s aim to increase the accumulation of EVs in the myocardium and discuss how this field can be elevated.

## Targeted delivery of extracellular vesicles to the heart

**FIGURE 1:**
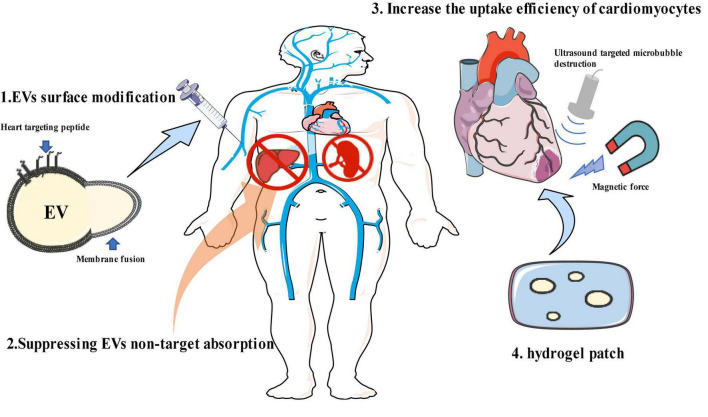
Strategies for enhancing extracellular vesicle (EV) heart-targeting efficiency. (1) EV surface modification; (2) suppressing EV non-target absorption; (3) increasing the uptake efficiency of target cells; (4) hydrogel patch. This figure contains modified images from Servier Medical Art (https://smart.servier.com) licensed by a Creative Commons Attribution 3.0 Unported License.

Most naturally secreted EVs show limited tropism to specific cell types. Thus far, four major types of strategies aimed at enhancing target efficiency have been described, including modifying EV surface to improve heart targeting, suppressing the non-target absorption, increasing the uptake efficiency of target cells, and employing the hydrogel patch (Figure 1). The improvement of effective targeting and delivery methods for EVs will lead to a paradigm shift in the development of more effective, more economical, and less-invasive treatments for many types of cardiovascular diseases.

## EV surface modification to improve heart targeting

Extracellular vesicles are internalized by target cells *via* membrane fusion, phagocytosis, micropinocytosis, and endocytosis (71). EV binding and uptaking differ between deriving cells and target cells (74). The surface molecules of EVs, such as tetraspanins, integrins, proteoglycans, and lectins, play a crucial role in these processes (17). Since differential protein expression has been documented between normal and ischemic myocardium, (75, 76), the principal approach to confer myocardium-targeting specificity to EVs is through surface modification. EVs engineered to over-express specific surface peptides with ischemia myocardial selectivity have demonstrated improved targeting performance. Through the application of phage display and *in vivo* biopanning techniques, which are widely used for the identification of cell targeting peptides (CTP), many myocardium homing peptides have been found and verified, and these peptides can target ischemic myocardium (77). The coding sequences of CSTSMLKAC are the most frequently utilized myocardium-targeting peptide (77–82). The examples of EV surface modification for myocardial targeted delivery are shown in Table 1.

**TABLE 1:** EV surface modification for heart targeting.

Surface modification strategy	Surface modification method	EV source	Targeting peptide	Animal model	Treatment outcome	References
Biological	Fused the CTP peptide to the Lamp2b protein by introducing a plasmid into HEK 293 cells	HEK 293 cells	APWHLSSQYS RT	MI mice	The delivery of EVs to the hearts was increased by 15% than control *in vivo* and 16% *in vitro*	(83)
Biological	Molecular cloning and lentivirus packaging to engineer Lamp2b fused with CTP peptide	BMSCs	CSTSMLKAC	MI mice	Modificated EVs were increasingly accumulated in the ischemic heart area	(82)
Biological	Engineered CDCs to express Lamp2b, which was fused to a CTP peptide by Lentivirus	Cardiosphere-derived cells	WLSEAGPVV TVRALRG TGSW	MI mice	Improved EVs uptake by cardiomyocytes *in vitro* and *in vivo*, decreased cardiomyocyte apoptosis	(84)
Chemical	Conjugated Exo with CTP peptide by bio-orthogonal chemistry	BMSCs	CSTSMLKAC	MI mice	Modificated EVs showed specific targeting to the ischemic lesions in the injured heart	(78)
Chemical	Conjugated EVs with CTP peptide through a DOPE-NHS linker	Cardiosphere-derived stem cells	CSTSMLKAC	MI rat	Increased retention of the EVs within heart sections *ex vivo* and *in vitro*	(79)
Chemical	Embed EV surfaces with an anchor conjugated to streptavidin	Cardiosphere derived cells	CSTSMLKAC	MI rat	Enhanced uptake in cardiac fibroblasts, myoblasts and ischemic myocardium	(80)
Chemical	Conjugated EVs with CTP peptide through a DOPE-NHS linker	human plasma EV	CSTSMLKAC	MI mice and dog	Improved cardiac retention of EVs in mice and dogs	(81)
Physical	Mixed EVs and platelet membrane and extruded them through filters	MSCs	N/A	MI mice	The uptake rate of platelet-membrane-hybrid exosomes that is 2–3 folds higher than that of control exosomes by endothelial cells, and 5–8 folds higher by cardiomyocytes	(85)
Physical	Mixed monocyte membrane vesicles and EVs and extruded with polycarbonate membrane	BMSCs	N/A	MI mice	Exhibited a high targeting efficiency for injured myocardium and improved therapeutic outcomes in cardiac function	(86)

EVs, extracellular vesicles; CTP, cell targeting peptide; BMSCs, bone marrow mesenchymal stem cells; MSCs, mesenchymal stem cells; MI, myocardial infarction.

Surface modification can be achieved through biological, chemical, or physical methods. For biological modification, by using gene transfer vectors, such as adeno-associated viruses (AAVs) or lentiviruses, to encode the sequences of myocardium-targeting peptide incorporated into the EV-donor cells genome, the host cells are genetically modified to express a myocardial homing peptide. This myocardial homing peptide is then fused with transmembrane proteins that are expressed on the surface of EVs (87). The lysosome-associated membrane glycoprotein 2B (LAMP-2B), which belongs to the lysosomal-associated membrane protein (LAMP) family and is expressed on EV membrane, is the most commonly used membrane protein (82–84, 88, 89). It comprises an N-terminal extramembrane domain and a C-terminal transmembrane domain, and the targeting peptide can be fused with the N-terminal extracellular domain of LAMP-2B to achieve a targeting effect.

By introducing vectors encoding cardiac-targeting peptide (CTP)-Lamp2B into HEK 293 cells, Kim et al. (83) generated CTP-expressing EVs. The *in vitro* and *in vivo* delivery efficiencies of the CTP-expressing EVs to the hearts were increased. Compared with blank EVs, a significant increase in targeting ischemic myocardium was observed in the myocardium-targeting peptide-engineered EVs, resulting in remarkable suppression of inflammation and cardiomyocyte apoptosis, enhanced angiogenesis, and reduced infarct size in a mouse MI model. A similar approach was employed by Mentkowski et al. (84), and they engineered cardiosphere-derived cells (CDCs) to express Lamp2b, which was then fused to a cardiomyocyte-specific peptide WLSEAGPVVTVRALRGTGSW. The EVs isolated from those engineered CDCs expressed cardiomyocyte-specific peptide on the surface and resulted in higher cardiac retention.

The surface of EVs can also be modified by a chemical approach after isolation. Compared with the biological modification method, chemical reactions are more rapid, efficient, and simple. Zhu et al. (78) conjugated EVs with an ischemic myocardium-targeted peptide by bio-orthogonal chemistry and found that modified EVs showed specific targeting to the ischemic lesions in the injured heart and exerted a marked cardioprotective function post-MI. Antes et al. (80) described an EV membrane anchoring platform termed “cloaking” to embed targeting moieties on EV surfaces. The cloaking system consists of three components: a DMPE phospholipid membrane anchor, a polyethylene glycol spacer, and a conjugated streptavidin platform molecule, to which any biotinylated molecule can be coupled for EV decoration. Vandergriff et al. (79) and Wang et al. (81) conjugated EVs with cardiac homing peptide through a dioleoylphosphatidylethanolamine N-hydroxysuccinimide (DOPE-NHS) linker and demonstrated enhanced homing efficiency of the injured heart and improved therapeutic outcomes.

Instead of genetically modifying EV donor cells or chemically modifying EVs, the physical approach of modifying the surface of EVs is membrane fusion. Zhang et al. (86) fused monocyte cell membranes with mesenchymal stem cell-derived EVs to generate monocyte mimetic EVs, which was performed by the fusion-extrusion method. These modified EVs were endowed with the inflammatory targeting features of monocytes, and these monocyte mimetic EVs could infiltrate injured myocardium from circulation. This approach exhibited a high targeting efficiency for injured myocardium and improved therapeutic outcomes in cardiac function. By extruding EVs and platelet membranes through 200 nm filters, Hu et al. (85) mixed platelet membranes and MSC-derived EVs to increase their macropinocytosis-mediated cellular internalization, as well as their ability to target the injured tissue. They found that platelet membrane modification of EVs significantly enhanced the cellular uptake of EVs by endothelial cells and cardiomyocytes and reduced the uptake of EVs by the mononuclear phagocyte system *in vivo*.

Surface peptide engineering conferred EVs with advanced targeting performance. However, the impacts of these methods on EV biological features are unknown. Whether this surface engineering performance will trigger side effects such as immunogenic activities or affect the normal functions of exosome membrane proteins still needs further investigation. Moreover, modification of the EV-deriving cells is time-consuming and exhausting, and these methods may damage EV membrane stability and integrity or lead to a low yield of EVs (90, 91). In addition, surface modification cannot load multiple surface peptides simultaneously (90). Another concern is that peptides displayed on the EV surface are unstable and may degrade. Hung et al. (92) demonstrated that membrane display by fusion with LAMP-2B may cause ligand loss. To protect peptides from degradation, engineered targeted peptide-Lamp2b fusion proteins with a glycosylation motif are experimentally feasible (83, 92).

## Suppressing EV non-targeted absorption

As exogenous vesicles in circulation, a challenge faced by EVs is the rapid hepatic clearance mediated by macrophages. Previous studies have shown that natural EVs can only be detected in the circulatory system for up to 1–3 h in normal mice after tail vein injection (63, 93, 94). Consequently, there has long been an interest in suppressing the non-specific uptake of EVs, prolonging the retention time of EVs in circulation, and, therefore, improving their chances of being absorbed by the heart.

CD47 is a transmembrane protein that enables cancer cells to evade clearance by macrophages through the initiation of a “don’t eat me” signal. By applying the EVs isolated from CD47 overexpressing MSCs, Wei et al. (95) prolonged the retention of CD47-EVs in the plasma, thus improving EV biodistribution in the heart.

Wan et al. (64) developed a strategy that uses blocking EVs to suppress the endocytosis of EVs by macrophages at first and then deliver therapeutic EVs to the heart. Blocking EVs were prepared by encapsulating EVs with siClathrin, which prevents EVs clathrin-dependent endocytosis by the macrophages in the liver and the spleen. This method significantly decreased the uptake of EVs by macrophages and improved the delivery efficiency of EVs to the heart. However, extra EVs are needed to produce blocking EVs in this method, which may lead to increased costs due to the low yield of EVs.

Suppressing the monocyte/macrophage or reticuloendothelial system to promote EVs absorption by the heart can be termed as “passive targeting,” which means after being injected into the circulation, EVs can avoid non-specific entrapment by the reticuloendothelial system and concentrate in the heart. The key dilemma in this strategy is that if other lesions were present, such as tumors, these lesions may competitively uptake EVs *in vivo* (96).

## Increasing the uptake efficiency of target cells

Once EVs are distributed to the desired tissues or organs, the increase in cell-specific uptake is another challenge. Targeting EVs to the heart is difficult, partly because the intact endothelial barriers block the entry of EVs, so promoting the uptake efficiency of cardiomyocytes is a practical approach for EV delivery. It has been reported that endocytosis is the major pathway of EV uptake (72, 97). Although other uptake routes also exist (71, 98, 99), including phagocytosis, membrane fusion, and micropinocytosis, the mechanism by which these processes are regulated remains unclear.

Ultrasound-targeted microbubble destruction (UTMD), which means burst gas-filled microbubbles under the assistance of ultrasound, can enlarge the capillary gaps and induce transient pores on cell membranes to promote the endocytosis of EVs (100–102), thus transiently enhancing the uptake of EVs by the target tissue. Particularly, when combined with ultrasound, the delivery of EVs can be controlled and enhanced in cardiomyocytes. Sun et al. proved that UTMD can significantly increase the EVs endocytosis by cardiomyocytes (65, 103). Though UTMD is an efficient and controllable targeting technique, high ultrasound pressures and corresponding microbubble generation may induce adverse effects (103). Therefore, the key to success is to optimize UTMD settings.

Alternatively, another study has used a magnet and pH-based method to improve the cardiomyocyte targeting specificity. Liu et al. (104) constructed pH-responsive magnetic nanoparticles decorated with two types of antibodies, which bind either to CD63 antigens on the surface of EVs or to myosin-light-chain surface markers on injured cardiomyocytes. These nanoparticles captured circulating EVs *in vivo* and the magnetic field guided nanoparticles accumulating in the heart; then, under the acidic pH of the injured myocardium, the EVs were released. This approach improved cardiac functions by circulating EVs. Concern about this method is that the toxicity of magnetic nanoparticles needs to be further studied; moreover, the magnetic force may be hard to reach the heart in obese patients.

## Employing hydrogel patch

Constant and fast blood flow also prevents EVs from accumulating in the heart. The strategy of encapsulating EVs in hydrogels prolongs EVs retention and uptake in the heart. In addition to *in situ* delivery, the special strength of hydrogel is the capacity to continuously release EVs. In this context, a variety of biomaterials can be used to produce functional hydrogels that reduce fibrosis, suppress inflammatory reactions, and promote cardiac function recovery (93, 105–109). Examples of EV hydrogel applications in MI treatment are shown in Table 2.

**TABLE 2:** Summary of EV hydrogels for MI treatment.

Composition of hydrogel	EV source	Animal model	Outcome	References
Adamantane-modified and β-cyclodextrin-modified hyaluronic acid hydrogel	endothelial progenitor cells	MI rat	The hydrogel steady released EVs for over 21 days	(107)
Cardiac protective peptides, matrix metalloprotease-2 (MMP-2) degradable sequence and hydrogel	human umbilical cord mesenchymal stem cells	MI rat	EVs encapsulated in the hydrogel can still be detected after 21 days	(105)
Gelatin and Laponite^®^	human adipose-derived stem cells	MI rat	The hydrogel prolonged the retention of secretome at the injection site	(108)
Collagen gel-foam mesh	cardiomyocytes derived from induced pluripotent stem (iPS) cells	MI rat	The hydrogel could slowly release EVs at least for 7 days after *in vivo* implantation	(93)
Self-assembling peptides hydrogels with bioactive peptides	BMSCs	MI rat	EVs alone or in conjunction with hydrogel improved cardiac function	(109)
Sodium alginate and calcium alginate	Dendritic cells	MI mice	Prolonged the retention of EVs 10–12 days *in vitro* and 14 days *in vivo*	(106)
Gelatin, methacrylic anhydride, and photoinitiators	BMSCs	MI mice	EVs remained visible in the cardiac tissue after 48 h	(110)

EVs, extracellular vesicles; BMSCs, bone marrow mesenchymal stem cells; MI, myocardial infarction.

Laponite^®^ is a synthetic smectite clay that is commercially available in pharmaceutics. By mixing Laponite ^®^ with the gelatin solution, Waters et al. (108) designed a nanocomposite hydrogel that can provide prolonged retention of stem cell-derived secretome at the myocardial infarction site. Zhang et al. (106) incorporated dendritic cell-derived EVs with alginate hydrogel and found prolonged retention of EVs *in vitro* and *in vivo*, resulting in a significant improvement of cardiac function after MI in mice. Han et al. (105) incorporated cardiac protective peptides and matrix metalloprotease-2 (MMP-2) degradable sequence with a novel injectable self-assembled hydrogel, which was used to encapsulate EVs. Compared with EVs alone, this mixture of hydrogel showed better cardiac function with reduced inflammation, fibrosis, and apoptosis. Liu et al. (93) placed an engineered collagen-based hydrogel patch on the myocardial tissue, and the EVs were capable of slowly releasing at least for 7 days after *in vivo* implantation. In another study, Chen et al. (107) produced a shear-thinning hydrogel that could steadily release EVs for over 21 days. A notable study in this area comes from Firoozi et al. (109), and they found that adding extra cardioprotective bioactive peptides in hydrogel could not further improve the therapeutic outcome of EVs. However, this observation is worthy of further investigation.

Although hydrogel patches can provide sufficient storage of EVs, huge obstacles to further application still exist because of their strong dependence on intramyocardial injection. To avoid secondary tissue injury by intramyocardial injection, Tang et al. (110) demonstrated an injection-free delivery approach by spraying a mixture of EVs, gelatin methacryloyl precursors, and photoinitiators, followed by visible light irradiation to form a cardiac patch covering the surface of the heart. This approach results in an enhanced retention rate of EVs and improved therapeutic effects. However, as thoracotomy is inevitable in this method, it is an invasive treatment too.

## Summary and perspective

Cardiovascular disease is a leading cause of death worldwide, and novel therapeutic strategies such as EVs demonstrate a promising future. Minimizing the invasive operation and improving the myocardial targeting delivery are the keys to successful clinical translation. Challenges in the efficient delivery of EVs to the heart can be mainly summarized into three aspects: First, naturally derived EVs showed low myocardial targeting efficiency; second, rapid uptake of EVs by non-targeted tissues; third, the compacted structure of myocardial tissue and constant fast blood flow in the heart prevents EVs uptake by the heart. To address these problems, four strategies have been reported so far: (1) modify EV surface to endow them with the property of targeting the heart. (2) Suppress the non-specific uptake of EVs and prolong the retention time of EVs in circulation. (3) Increase the uptake efficiency of cardiomyocytes. (4) Encapsulate EVs into hydrogels. The targeting effects and cardioprotective vary depending on the targeting strategy as well as the source and content of EVs. Therefore, it is challenging to determine which approach is better due to the lack of adequate controls, comparable doses, and dosing frequency. Albeit, each method demonstrated improved EV uptake and retention in the heart and led to improvements in therapeutic outcomes.

In future studies, combining these targeting strategies will be a feasible direction. For example, combining “passive targeting” with surface modification of myocardial homing peptides may dramatically boost the targeting effectiveness. Furthermore, it has been widely reported that exposure to ultrasonic irradiation could trigger a short-time, high-dose release of drug encapsulated in a hydrogel on demand (111–114). Thus, it is rational to assume that combining ultrasound stimulation and hydrogel may control the delivery timing of EVs to the heart and enhance their uptake efficiency. In another study, Wang et al. (115) fabricated a targeting peptide and ultrasound-responsive nanodrug in cancer therapy, and this approach is also worth trying in EV targeting. Finally, it should be noted that the strategies employed for EV heart targeting can also be applied in other fields such as tumor targeting (116) and brain targeting (117).

Extracellular vesicles are regarded as an effective therapeutic modality for the treatment of CVD, although current research outputs remain in their infancy and distant from clinical translation. A better understanding of the internalization and trafficking of EVs *in vivo*, as well as their biogenesis, heterogeneity, and interaction with receiving cells, is required to maximize their therapeutic effectiveness. Moreover, the targeting effect of EVs has only been confirmed in small laboratory animals, which is partly due to the limited output of EVs, and systematic non-human primate experiments are still required before translation into humans. Despite all these challenges, with the continuous development of targeting delivery strategies, this field demonstrates a promising future.

## Author contributions

XY: conceptualization, investigation, and writing—original draft. L-HJ: conceptualization, funding acquisition, resources, supervision, writing—review and editing. Both authors contributed to the article and approved the submitted version.
